# A rare combination of pheochromocytoma and gastrointestinal stromal tumour in a patient with neurofibromatosis 1 syndrome—a case report

**DOI:** 10.1186/s40792-015-0107-4

**Published:** 2015-10-14

**Authors:** P S Jayalakshmy, A. Anish Mohan, Rajesk K. Kumar, P. Junaina Beevi

**Affiliations:** Government Medical College, Thrissur, Kerala India; Parijatham, Royal Avenue, Near Attore Road Bus Stop, Kuttur. P.O., Thrissur, PIN-680013 Kerala India

**Keywords:** Brown fat, Clear cell nodules, Gastrointestinal stromal tumour (GIST), Inherited syndromes, Neurofibromatosis 1 (NF1), Pheochromocytoma

## Abstract

Neurofibromatosis 1 is a rare inherited autosomal dominant syndrome. It comprises 90 % of neurofibromatosis cases. These patients may develop various types of tumours in early age, especially multiple neurofibromas with a high risk of developing malignant peripheral nerve sheath tumours. Other tumours can also develop like pheochromocytoma, optic nerve and brain stem gliomas, carcinoids and rarely gastrointestinal stromal tumours. A combination of pheochromocytoma and gastrointestinal stromal tumour is very rare. Only a few cases have been reported. Here, we are reporting a case of NF1 syndrome with a combination of pheochromocytoma and gastrointestinal stromal tumour with additional findings of multiple clear cell nodules and brown fat in the periadrenal connective tissue.

## Background

Neurofibromatosis (NF) is a rare autosomal dominantly inherited syndrome with an incidence of 1 in 3000 live births [1]. Of the two types, neurofibromatosis 1 and 2 (NF1 and NF2, respectively), NF1 comprises 90 % of the cases. The NF1 gene encoding the protein neurofibromin has been localized to chromosome 17q11.2, and the NF2 gene encoding the protein merlin is on chromosome 22. NF shows complete penetrance but variable expressivity. Apart from multiple neurofibromas, many other neoplasms may be seen in such patients. One among them is the adrenal tumour pheochromocytoma. Gastrointestinal stromal tumour (GIST) is also described in NF1 patients, but a combination of both these is very rare with only about 15 cases documented till 2014 in the English literature. Gorgel et al. [2] have described three NF1 patients with this combination. We report such a case with additional findings of multiple clear cell nodules and brown fat in the periadrenal connective tissue.

## Case report

**Fig. 1:**
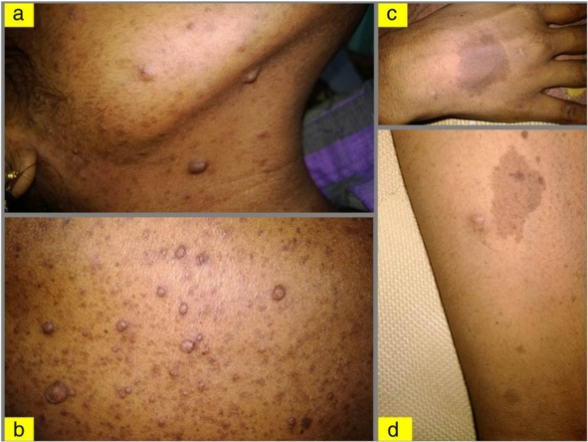
Clinical photograph of the patient. **a**, **b** Multiple neurofibromas. **c**, **d** Café-au-lait spots more than 1.5 cm size as per criteria in adults

A 26-year-old female presented with complaints of flank pain, headache and hypertension detected recently. On clinical examination, the patient was found to have neurofibromatosis 1. The patient had multiple café-au-lait spots, more than six which were more than 15 mm in size, and multiple nodules of neurofibroma all over the body (Fig. 1a–d). Axillary and inguinal freckling was present. On further enquiry, patient revealed that her mother is also having similar lesions in the body.

**Fig. 2:**
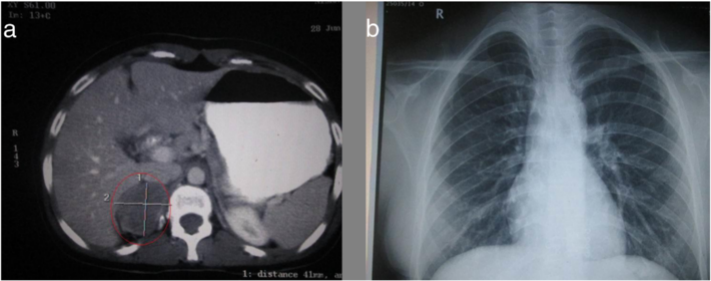
**a** CT abdomen—note the mass in the right adrenal area. **b** Plain x-ray chest—within normal limits

Computerised tomography studies revealed a right adrenal mass (Fig. 2a). Routine x-ray of the chest was normal (Fig. 2b).

Complete blood counts, blood glucose, liver, renal and thyroid function tests were within normal limits. Along with routine investigations, tests for the functional status of adrenal mass were done. The plasma metanephrine level which is the metabolite of epinephrine was significantly elevated (Table 1).

**Table 1:** Investigations for functional status assessment of adrenal tumour

Patient value	Blood/urine tests	Normal value
2.5 pg/dl	Plasma adrenaline (epinephrine)	<50 pg/dl
90.30 pg/dl	Plasma noradrenaline (norepinephrine)	110–410 pg/dl
5.1 mg/dl	Urine 24 h VMA	1.8–7.2 mg/dl
326 pg/dl	Plasma metanephrine (ELISA)	<90 pg/dl
10.89 μg/dl	Serum cortisol	5–25 μg/dl

The patient underwent right adrenalectomy under general anaesthesia. During surgery, two small extra luminal nodular masses were seen in the jejunal wall which were also excised.

We received in the pathology department the right adrenalectomy specimen and excision specimen of the jejunal wall nodules.

**Fig. 3:**
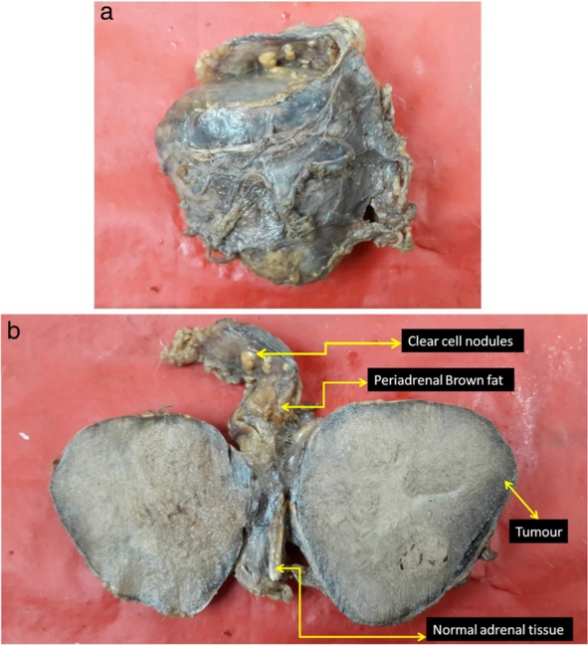
**a** Gross specimen of right adrenalectomy—pheochromocytoma. Note the yellow nodules in the fat. **b** Cut section of pheochromocytoma with brownish/tan colour of the tumour. Normal adrenal tissue is seen on one side. Three yellow nodules are visible in the periadrenal fat

Right adrenalectomy specimen measured as 6.5 × 6.5 × 2.5 cm and weighed 75 g (Fig. 3a). The cut section showed an encapsulated grey-white growth measuring 5.5 × 6 × 2 cm. The periphery showed part of normal adrenal tissue and fat. Another interesting finding was the presence of three small yellow nodules, the largest measuring 0.8 cm in the periadrenal fat (Fig. 3b).

**Fig. 4:**
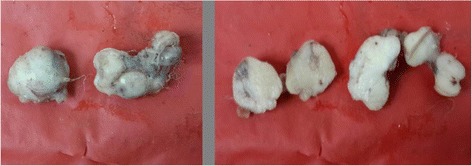
Gross and cut sections of two nodules of jejunal GIST

The jejunal wall growth excision specimen showed two nodular grey-brown tissue; the larger measured 2.5 × 1.5 × 1 cm, and the smaller one measured 2 × 1.5 × 1 cm. The cut section of both showed a grey-white appearance with faint lobulations (Fig. 4).

**Fig. 5:**
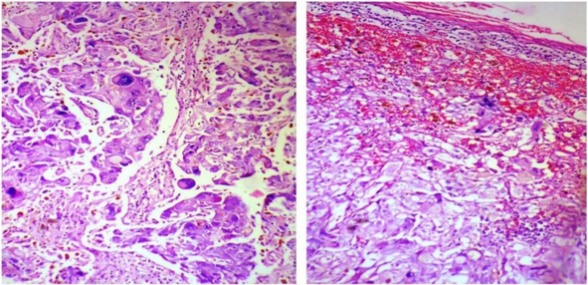
Histology of pheochromocytoma—different areas (H&E ×400). Highly pleomorphic cells with interspersed vascular channels. Adrenal tissue is seen in the periphery

**Fig. 6:**
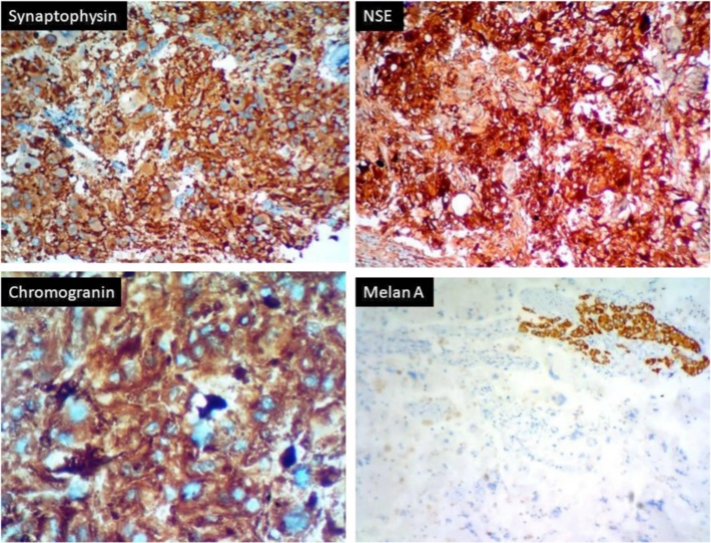
IHC stains of pheochromocytoma—diffuse strong positivity for NSE, synaptophysin and chromogranin

**Fig. 7:**
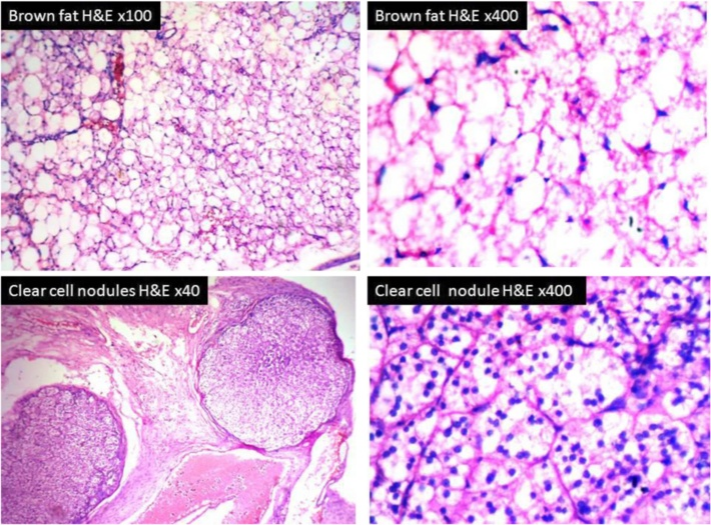
Brown fat and nodules of clear cells (H&E)

Microscopy of the adrenal mass showed normal adrenal tissue in the periphery with a circumscribed neoplasm showing histological features of pheochromocytoma (Fig. 5). Immunohistochemical staining (IHC) showed a diffuse strong positivity for chromogranin, NSE and synaptophysin in the tumour cells and negativity for melan A (Fig. 6). The yellow nodules histologically showed a circumscribed collection of clear cells. The fat adjacent to the tumour showed features of brown fat histologically (Fig. 7).

**Fig. 8:**
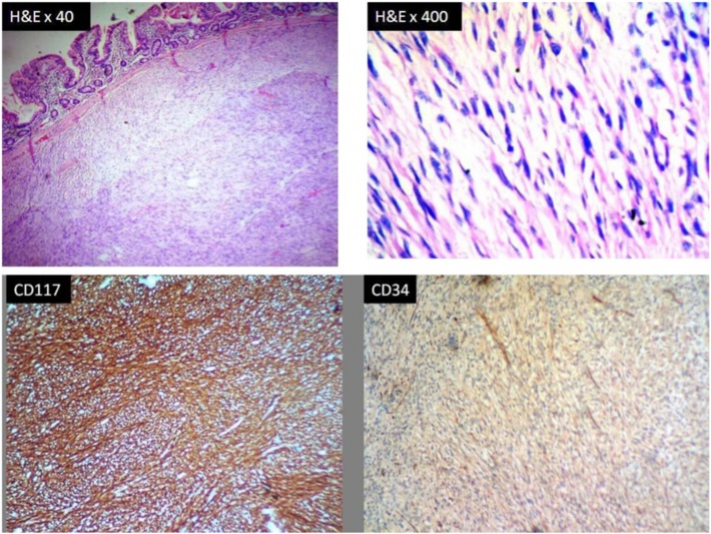
Showing lesion in the submucosa of the jejunum with spindle cells arranged in a fascicular pattern (H&E). IHC staining CD117 showing diffuse strong positivity. CD34 showing negativity in tumour cells.

Nodules from the jejunal wall showed a spindle cell neoplasm arranged in sheets and fascicles. IHC of this was positive for CD117 and negative for CD34. SMA was negative confirming the diagnosis of gastrointestinal stromal tumour (Fig. 8).

### Discussion

Neurofibromatosis (NF) is a familial syndrome with complete penetrance and variable expressivity. There are two types—NF1 and NF2—according to the specific molecular defect. The present case was diagnosed clinically as NF1 because four of the seven criteria of the National Institute of Health (NIH) consensus conference of 1987 (http://consensus.nih.gov/1987/1987Neurofibramatosis064html.htm) were met in this case, namely, (1) six and more café-au-lait spots more than 15 mm in size were present, (2) many neurofibromas seen in the body, (3) freckling in the axillary/inguinal regions were noted and (4) the mother diagnosed to have NF1. (Two or more of the seven defined criteria are needed for diagnosis of NF1.)

Pheochromocytoma occurs in less than 1 % of NF1 patients [3]. The tumour presents clinically with features of hypertension. Our patient presented with hypertension and had headache and flank pain. The tumour can be clearly delineated by imaging studies. In two of the three cases in Gorgel et al.’s case report, the adrenal tumour was bilateral [2]. In the present case, only the right adrenal was involved by the tumour; the left adrenal was normal in imaging studies.

Other more common hereditary conditions with pheochromocytoma are the von Hippel-Lindau syndrome (vHL), multiple endocrine neoplasia syndrome type 2A and 2B (MEN 2A and 2B) and familial paraganglioma syndrome. Other associated features of MEN and vHL syndromes were not present in this patient. No paragangliomas were detected in extra adrenal sites ruling out a paraganglioma/pheochromocytoma syndrome. Carney triad is also known to present with adrenocortical adenomas, along with pheochromocytoma and GIST. In most cases, prototypical lesions of the triad-pulmonary chondromas are also seen. But in the present case, there was no lung lesion.

Reported gastrointestinal (GI) manifestations in NF1 range from GI benign and malignant neural tumours, neuroendocrine tumours and adenocarcinoma especially of the ampullary region and GIST [4]. The incidence of GISTs among NF1 patients varies from 3.9 to 25 %, while the overall ratio of NF1 among GIST patients is about 6 % [5]. A combination of pheochromocytoma and carcinoid has been reported in NF1 patients. But the coexistence of pheochromocytoma and GIST is very rare. Only a few cases have been reported in the English literature. Gorgel et al. proposed that the activation of the *Ras* pathway responsible for neurofibroma formation in NF1 patients also causes Cajal cell proliferation, leading to GIST development [2].

The GISTs in NF1 patients are usually multiple in number and do not cause any symptoms, hence, are detected incidentally. In our case, there were two lesions in the jejunal wall and were detected incidentally during the surgical procedure for adrenal tumour.

In NF1 patients, more than 300 mutations in the NF1 gene have been identified. Zero to 15 % show identifiable somatic CKIT or platelet-derived growth factor receptor-alpha (PDGFRA) mutations in GIST. We had done IHC for CKIT (CD117) in our case and found it to be positive. Two syndromes, Carney triad and Carney-Stratakis syndrome, show succinate dehydrogenase (SDH)-deficient GIST, but markers for this are not available in our centre. The majority of NF-GISTs are benign but can become malignant. In our case, it was benign according to the criteria.

Another interesting finding in our case was the presence of three clear cell nodules in the periadrenal connective tissue. Clear cell collection has been described as a degenerative change in pheochromocytoma and was first reported by Ramsay et al. [6] in a 27-year-old man with bilateral adrenal pheochromocytomas having a family history of von Hippel-Lindau disease. They have demonstrated the nature of these cells as similar to pheochromocytoma cells by electron microscopy examination. But, in their report, the clear cell nodules were seen within the tumour itself. In our case, the yellow nodules were noted in the periadrenal fat. The second case report was by Unger et al. in an 18-year-old woman with a family history of multiple endocrine neoplasia type IIA [7]. No other reports have been seen in English literature even on extensive search. Hence, this may be the third case being reported with this finding in association with pheochromocytoma. It is still not clear in our case whether these clear cell nodules are lipid degeneration of pheochromocytoma, adrenocortical-cell-type nodules or lipid degeneration of periadrenal fat. In our centre, electron microscope is not available for further examination.

In this case, periadrenal fat showed the features of brown fat. Adrenal pheochromocytoma and brown fat association has been first described by Melicow in 1957 [8]. According to Medeiros et al., it is a topic of debate whether the pheochromocytoma induces the development of brown fat or if it is a coincident finding [9]. Rona [10] described two patients with adrenal pheochromocytomas and large masses of brown fat, both adjacent to and distal from the tumour. Medeiros et al. [11] reviewed 60 cases of pheochromocytoma and found brown fat in association with these tumours in 73 % of cases.

## Conclusions

A rare combination of pheochromocytoma and GIST along with multiple clear cell nodules and brown fat in the periadrenal connective tissue is being reported in a patient with NF1 syndrome. Whenever a young patient presents with a combination of lesions, a syndrome should be suspected, and the patient should be screened thoroughly for the associated lesions so that an early diagnosis can be made and the patient can be saved by appropriate management.

## Consent

Written informed consent was obtained from the patient for publication of this case report and any accompanying images. A copy of the written consent is available for review by the Editor-in-Chief of this journal.
